# Mechanical behavior of endodontically treated molars restored using two restorative approaches and two CAD/CAM materials: an in-vitro study

**DOI:** 10.1186/s12903-026-07835-4

**Published:** 2026-09-05

**Authors:** Ahmed Adel Naguib Khater, Ahmad Khaled Abo El Fadl, Ingy Nouh

**Affiliations:** Fixed Prosthodontics Department, Faculty of Dentistry, Ain Shams University, Cairo, 3753450 Egypt

**Keywords:** Endodontically treated teeth, CAD/CAM restorations, Fracture resistance, Endocrowns, Overlays, Chewing simulation

## Abstract

**Background:**

Restoration of endodontically treated teeth (ETT) with significant coronal loss poses a challenge in clinical dentistry. Advances in CAD/CAM technology and biomimetic materials have introduced minimally invasive approaches, such as endocrowns and overlays, which can replace traditional post-core and crown approach.

**Aim:**

The aim of this in-vitro study was to comparatively assess the mechanical behaviour of two restorative designs (endocrowns and overlays) fabricated from two CAD/CAM materials in endodontically treated mandibular molars.

**Materials and methods:**

Thirty-six endodontically treated lower molars (*n* = 36) were split into two main groups based on restoration type (*n* = 18): endocrowns and overlays. Each group was further subdivided into two subgroups based on the CAD/CAM material (*n* = 9): lithium disilicate (Tessera) and indirect composite (Crios). Restorations were fabricated and bonded to their respective specimens following a strict bonding protocol with an adhesive resin cement (Bisco self-adhesive). Thermo-mechanical aging (Chewing Simulator CS-4, SD Mechatronic) was conducted to simulate oral conditions, followed by a universal testing machine's evaluation of fracture resistance. The test was stopped after fracture. Fractured parts were analysed to determine mode of failure. Data were analyzed using two-way ANOVA followed by Tukey’s honestly significant difference (HSD) test for pairwise comparisons (α = 0.05).

**Results:**

All specimens survived the 5,000 chewing cycles, simulating early intraoral service. Fracture resistance test showed significantly higher fracture resistance for overlays (2048.46 ± 664.63 N) than endocrowns (1234.54 ± 338.50 N) (*p* = 0.002). Crios overlays exhibited the highest fracture resistance (2384.60 ± 631.10 N), significantly outperforming Tessera overlays (1712.33 ± 559.59 N) (*p* = 0.043) and endocrowns (1354.20 ± 346.88 N) (*p* = 0.004). No substantial material change was seen in endocrown restorations (*p* = 0.445).

**Conclusion:**

Overlay restorations and indirect composite material (Crios) provide superior resistance to fracture compared to lithium disilicate overlays and endocrowns and may represent a reliable restorative option for endodontically treated molars.

## Introduction

The restoration of endodontically treated teeth (ETT) with significant coronal loss remains a topic of ongoing debate in dentistry, particularly regarding the optimal restorative design and material selection to balance fracture resistance and tooth preservation [1–5]. Traditionally, such teeth were predominantly restored using conventional crowns supported by post and core systems [1, 2]. This approach was long believed to strengthen the rest of tooth structure [6]. Nevertheless, the necessity of post insertion often weakens the teeth due to the extensive dentin removal and invasive preparation required during the procedure.

Advancements in ceramic materials and adhesive dentistry have revolutionized restorative techniques, reducing the reliance on post and core systems [7–10]. The development of etchable ceramics and adhesives has facilitated the use of adhesive partial-coverage restorations, eliminating the need for the extensive preparation required for full-coverage crowns [11]. Innovative designs, such as endocrowns and overlays, have gained prominence for ETT restoration. Literature supports monolithic endocrowns use, which achieves retention and eliminates the need for posts based on pulp chamber preparation [12–15]. Similarly, overlays refers to a partial-coverage restoration replacing the occlusal surface and involved cusps, bonded adhesively without full-crown coverage constructed with bioadhesive bases have been suggested to replicate the biomechanical properties of natural teeth [15].

Despite these advancements, ETT restoration with non-ideal characteristics, such as minimal remaining wall height, inadequate ferrule, or compromised tooth structure, remains inadequately addressed in the literature. There is limited evidence on the optimal methodologies or selection criteria for restoring severely decayed teeth, leaving clinicians with challenging decision-making scenarios [3].

Endocrowns which are corono-radicular monoblock restorations relying on adhesive bonding and macromechanical retention within the pulp chamber rather than complete external coverage of the tooth have demonstrated higher success rates in molars [4, 5]. Overlays describe partial-coverage restorations involving complete occlusal surface and cusp coverage without axial wall extension and may be considered an advanced form of an onlay [16].

Materials such as CAD/CAM blocks of indirect composites and lithium disilicate are widely used in fabricating endocrowns and overlays. Hybrid materials, which combine properties of both indirect composites and lithium disilicate, have emerged as a preferred option due to their balanced advantages [17].

Biomimetic dentistry, focusing on restoring teeth to their natural physical and biomechanical harmony, has seen significant advancements. This approach emphasizes using materials that mimic natural enamel and dentin, such as composites with a similar elastic modulus to dentin and ceramics with high hardness resembling enamel. These materials function cohesively as a bonded unit, promoting long-term restoration success [18].

Despite the extensive literature on restoring endodontically treated teeth, there remains limited evidence directly comparing restorative design and CAD/CAM material behavior within the same experimental framework, particularly under standardized thermomechanical aging conditions. Moreover, few studies have evaluated the combined influence of restoration design and material selection on fracture resistance and failure mode in mandibular molars with standardized MOD preparations and controlled pulp chamber dimensions. Therefore, the present study aimed to provide a factorial comparison of endocrowns and overlay restorations fabricated from two commonly used CAD/CAM materials (Tessera and Crios), offering clinically relevant insight into design–material interactions. The null hypothesis was that neither the restoration design nor the CAD/CAM material would significantly affect fracture resistance of endodontically treated mandibular molars.

## Material and methods

**Fig. 1:**
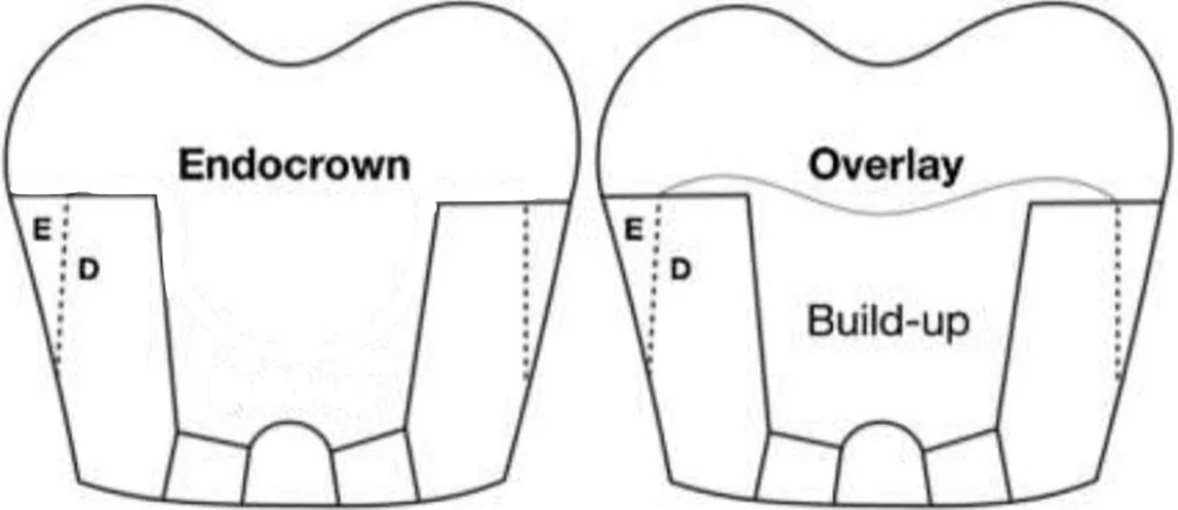
Schematic representation of endocrown and overlay restorations relative to pulp chamber build-up

In this in-vitro study thirty-six endodontically treated lower first molars (*n* = 36) were utilized and split into two main groups according to the restoration approach (*n* = 18): endocrowns and overlays. Each group was further subdivided into two subgroups according to the material (*n* = 9): lithium disilicate (Tessera) and indirect composite (Crios) over short fiber-reinforced composite core (Fig. 1).

### Sample size calculation

A priori sample size calculation was performed using GPower software (version 3.1.9.7; Heinrich Heine University, Düsseldorf, Germany). The calculation was designed to provide adequate power to test the null hypothesis that no difference exists between the tested groups regarding fracture resistance. Assuming an alpha (α) level of 0.05, a power of 80% (β = 0.20), and an effect size (f = 0.585) derived from previously published fracture resistance data, a total sample size of 36 specimens was required for a 2 × 2 factorial design. Accordingly, 18 teeth were allocated to each main group and further subdivided into four subgroups (n = 9 per subgroup). The sample size calculation was primarily powered to detect main effects, while interaction effects were considered exploratory.

### Preparation of specimens

This in-vitro study was conducted using previously extracted human teeth collected for reasons unrelated to the present research. No human participants were involved, and no personal or identifiable patient data were used. Ethical approval for the use of extracted human teeth was obtained from the Ethics Committee of the Faculty of Dentistry, Ain Shams University (FDASU-Rec: EM122231). Thirty-six permanent mandibular first molars extracted due to periodontal diseases were collected from the Department of Surgery, Faculty of Dentistry, Ain Shams University. The extracted molars were selected after stereomicroscopic examination (magnification × 10) to confirm they were sound, non-carious, and free from cracks, with fully formed apices. The depth of the pulp chamber varied between 5 and 7 mm, assessed using a periodontal probe. After being immersed in a solution of 5% formaline and saline for two hours, the teeth were rinsed and placed in distilled water to avoid drying out. Working lengths were set 1 mm short of the apical foramen using a size 10 K-file to estimate the canal lengths. Root canal treatment used the Protaper system (F2 for mesial, F3 for distal canals) with sodium hypochlorite irrigant. Gutta-percha and resin-based sealant were used for obturation, and excess material was removed with a condenser.

### Tooth preparation

All tooth preparations were standardized using a computer-aided design/computer-aided manufacturing (CAD/CAM) milling workflow. Each natural tooth was fixed to a CAD/CAM milling block and digitally scanned. Standardized MOD cavity preparations were digitally designed (Exocad GmbH, Darmstadt, Germany) according to predetermined dimensions; 8–10 degrees of internal taper, isthmus width of 4 mm, depth of pulp chamber of 5–7 mm according to natural anatomical variation and 2-mm flat occlusal reduction with a 90 degrees butt margin. The preparations were subsequently milled using a CAD/CAM milling machine (inLab MC X5, Dentsply Sirona, Charlotte, NC, USA) to ensure uniform preparation geometry across all specimens, following a methodology previously described by Chai et al. [19].

After milling, roundation of internal line angles and margin finishing were carried out manually using fine diamond burs (Intensive SA, Montagnola, Switzerland) to eliminate sharp line angles and reduce stress concentration, in accordance with the referenced protocol.

Teeth were then mounted in moulds made from polypropylene tubes (2.50 cm diameter, 6 cm height) filled with non-shrink epoxy resin. A surveyor ensured the teeth were positioned upright, centralized, and with the epoxy resin margin 3 mm below the cement-enamel junction, secured using pink wax.

For the overlay group, the pulp chamber was partially filled using a short fiber–reinforced composite (everX Posterior; GC, Leuven, Belgium). The material was applied in ≤ 2-mm increments, each increment was adapted using a plastic instrument to minimize void formation, and light-cured for 40 s using an LED curing unit (Elipar™ DeepCure-S; 3 M ESPE, St. Paul, MN, USA) according to the manufacturer’s instructions. The final fiber-reinforced layer was finished to achieve a standardized 2-mm pulp chamber depth. To avoid direct exposure of everX to the oral environment, a layer of packable composite resin was applied over the marginal ridges and light-cured [20].

### Sample grouping

Thirty-six lower molars were split into 2 groups (*n* = 18); group E restored by ceramic endocrown and group O restored by occlusal overlay over a fiber resin core. Each group was subdivided into two subgroups (*n* = 9) according to the material; subgroup T advanced lithium disilicate (Tessera, Dentsply Sirona, Charlotte, NC, USA) and subgroup C indirect composite (Crios, Coltène/Whaledent AG, Altstätten, Switzerland). Random allocation was performed using a computer-generated random number table by an investigator not involved in specimen preparation or testing (Table 1).

**Table 1:** Experimental Factorial Analysis

**Material/Design**	**Tessera**	**Crios**	**Total**
Endocrown (Group E)	TE (*n* = 9)	CE (*n* = 9)	**18**
Overlay (Group O)	TO (*n* = 9)	CO (*n* = 9)	**18**
Total	**18**	**18**	**36**

### Restoration fabrication

#### Scanning

Teeth were scanned from multiple directions using an Identica Blue scanner (Medit, Seoul, South Korea) to generate a precise 360-degree model. The model phase involved defining margins and setting the insertion axis to prevent undercuts and ensure proper fit.

#### Designing

In the design phase, an open CAD/CAM system comprising Exocad software (Exocad GmbH, Darmstadt, Germany) was used and ceramic endocrown and onlay thickness was verified.

#### Restoration fabrication

Restorations were fabricated using a VHF milling machine (VHF Camfacture AG, Ammerbuch, Germany). The process included five phases: in the administration phase, the restoration type, design technique, and material were selected in Exocad. Finally, the milling phase used Exocad software to guide the VHF milling machine in fabricating the restorations.

### Restoration checking, finishing and polishing

After milling, sprues/attachment points were removed, and overmilling irregularities and margin flash were finished using fine diamond burs. Internal line angles were refined to avoid stress concentration. Group T underwent crystallization firing in a ceramic furnace (Programat P310, Ivoclar Vivadent, Schaan, Liechtenstein) according to the manufacturer’s recommended firing cycle at a final temperature of 760 °C, with a holding time of 10 min under vacuum, prior to surface treatment and cementation.

Prior to cementation, the internal surfaces of the Tessera restorations were etched using 9% hydrofluoric acid gel (Porcelain Etch; Ultradent Products Inc., South Jordan, UT, USA) for 20 s, thoroughly rinsed with water, and air-dried. A silane coupling agent (Silane Primer; Ultradent Products Inc., South Jordan, UT, USA) was then applied according to the manufacturer’s instructions and allowed to react. The internal surfaces of the Crios restorations were air-abraded with 50-µm aluminum oxide particles at a pressure of 2 bar from a distance of approximately 10 mm for 10 s, followed by cleaning and air-drying.

Tooth surfaces were etched with 35% phosphoric acid gel (Ultra-Etch; Ultradent Products Inc., South Jordan, UT, USA) for 20 s, rinsed thoroughly, and gently air-dried. A universal adhesive (All-Bond Universal; BISCO Inc., Schaumburg, IL, USA) was applied to the tooth surfaces using a microbrush, gently air-thinned for 5 s, and light-cured for 20 s according to the manufacturer’s instructions.

All restorations were cemented using a self-adhesive resin cement (BISCO Inc., Schaumburg, IL, USA). The cement was applied to the intaglio surface of each restoration, which was then seated under finger pressure. Excess cement was removed, and the restorations were light-cured for 2 s to facilitate clean-up, followed by final polymerization using a high-intensity LED curing unit (Elipar™ DeepCure-S; 3 M ESPE, St. Paul, MN, USA). A glycerin gel was applied at the margins prior to final curing to prevent formation of an oxygen-inhibited layer.

### Thermomechanical aging and fracture resistance testing

**Fig. 2:**
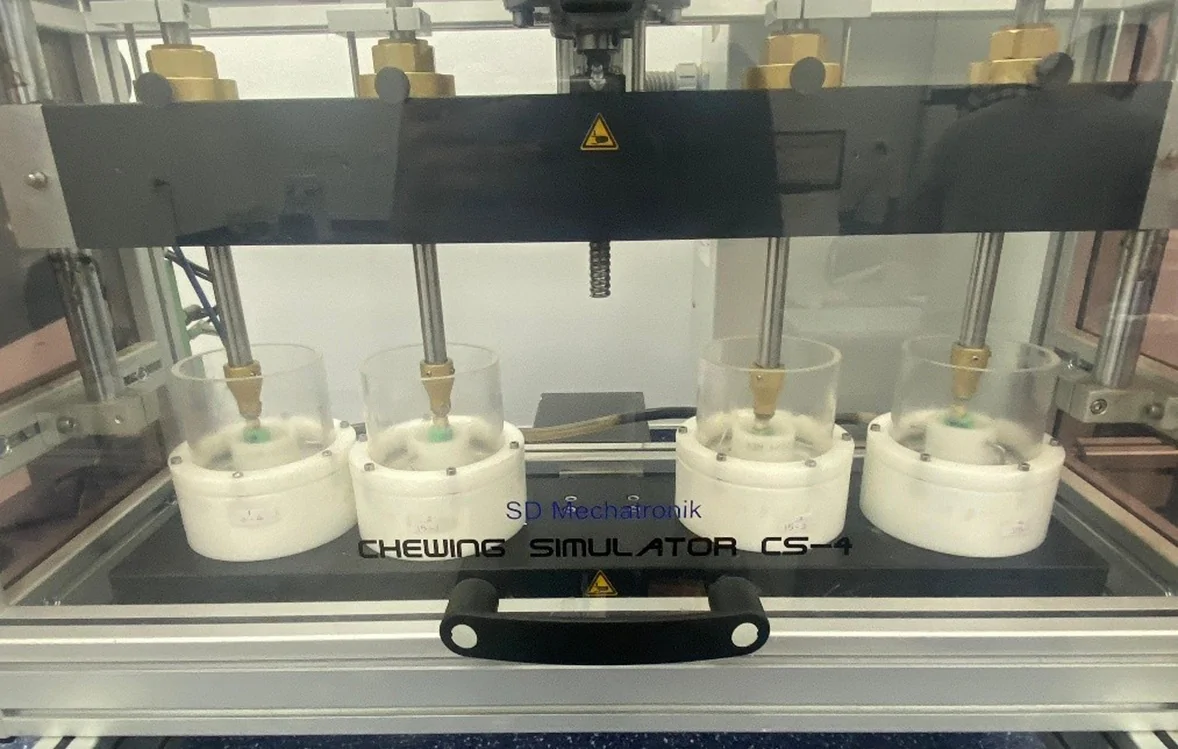
Chewing simulator for thermo-mechanical loading

Thermomechanical aging was performed using a chewing simulator (CS-4, SD Mechatronic, Feldkirchen-Westerham, Germany) to simulate early clinical service (Fig. 2). Specimens were subjected to 5,000 mechanical loading cycles at a load of 49 N and a frequency of 1.6 Hz, combined with thermocycling between 5 °C and 55 °C. Each thermal cycle consisted of a 20-s dwell time in each bath with a 10-s transfer time, resulting in an approximate cycle duration of 50 s. A stainless-steel spherical antagonist (6 mm diameter) was used to apply cyclic loading on the central fossa of each restoration. This type of loading allows repetitive cyclic forces on specimens to simulate the functional stresses experienced in the oral cavity during mastication. After aging, specimens were inspected for cracks/debonding and then tested to fracture under static loading [21, 22].

**Fig. 3:**
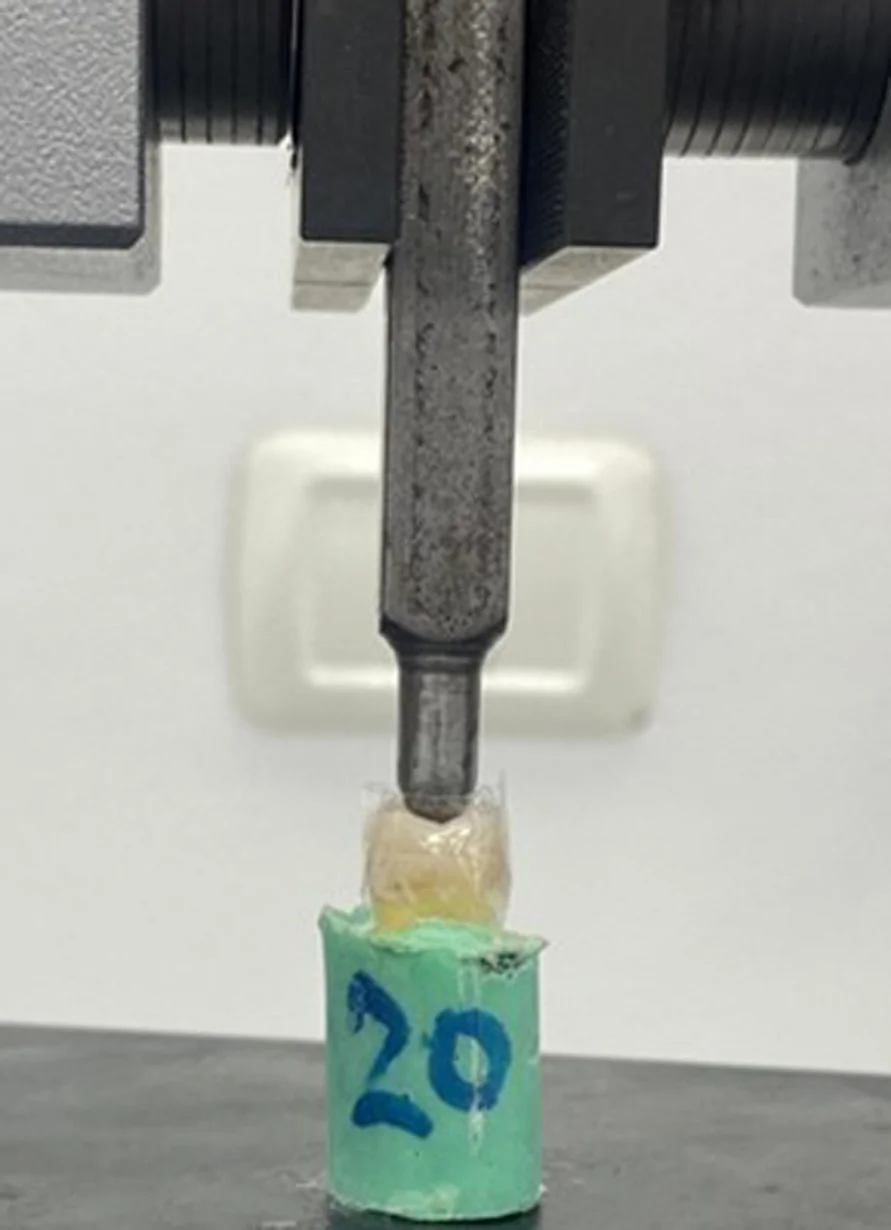
Universal testing machine for fracture resistance test

**Fig. 4:**
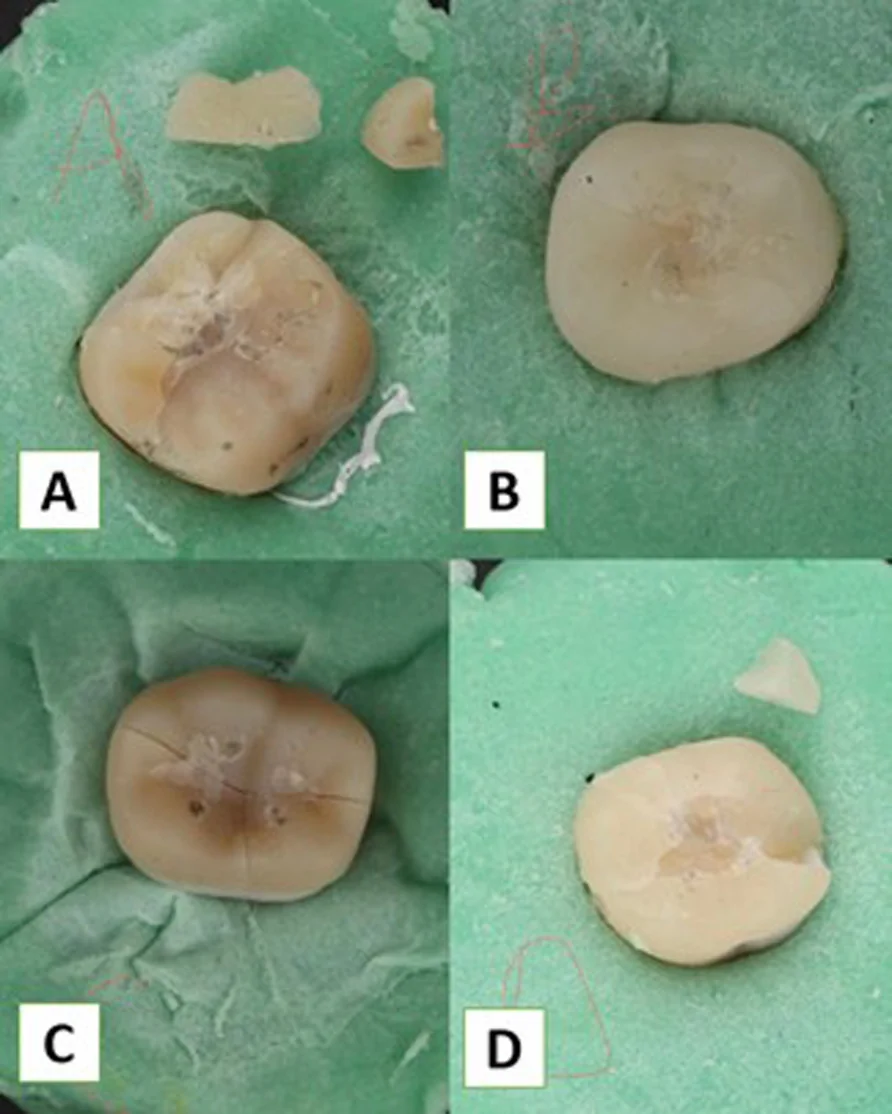
**A** TE shows cohesive adhesive failure without tooth structure, **B** TO shows cohesive failure without tooth fracture, **C** CE shows cohesive adhesive failure without tooth fracture, **D** CO shows cohesive failure with tooth fracture

The next step was to evaluate the fracture resistance using a universal testing machine (WDW-5TC, Shinae Corp., China). Until fracture occurred, specimens were subjected to vertical loading on the central fossa using a 6 mm steel ball at a thrust speed of 0.5 mm/min. Breaking loads were recorded in Newtons (N) based on sudden load drops and acoustic signals (Fig. 3). Each specimen was assessed for failure mode and analysed (Fig. 4).

### Statistical analysis

Categorical data were summarized as frequencies and percentages, whereas numerical data were expressed as means with standard deviations (SD), 95% confidence intervals (CI), and minimum and maximum values. Normality and homogeneity of variances were evaluated using the Shapiro–Wilk and Levene’s tests, respectively. Although the power of these tests is limited with small sample sizes, a two-way analysis of variance (ANOVA) was applied due to its robustness in balanced factorial designs.

Two-way ANOVA was used to assess the main effects of restoration design and CAD/CAM material, as well as their interaction. When significant effects were detected, pairwise comparisons were performed using Tukey’s honestly significant difference (HSD) test. Mean differences were reported with 95% confidence intervals to facilitate interpretation beyond p-values. A significance level of p < 0.05 was adopted. All analyses were conducted using R software (version 4.4.2; R Foundation for Statistical Computing, Vienna, Austria).

## Results

All specimens survived 5,000 cycles of chewing simulation first. Two-way ANOVA revealed a significant main effect of restoration design on fracture resistance (F(1,32) = 14.19, p = 0.002), indicating that restoration design significantly influenced fracture resistance values. The main effect of CAD/CAM material was not statistically significant (F(1,32) = 4.45, p = 0.051), suggesting comparable fracture resistance values between the tested materials when design was not considered. The interaction between restoration design and CAD/CAM material was also not statistically significant (F(1,32) = 1.00, p = 0.331), indicating that the effect of restoration design on fracture resistance was not dependent on the material used.

Post-hoc pairwise comparisons using Tukey’s honestly significant difference (HSD) test demonstrated that overlay restorations exhibited significantly higher fracture resistance than endocrowns (p = 0.002). Within the overlay design, Crios showed significantly higher fracture resistance than Tessera (p = 0.043). In contrast, no statistically significant difference was observed between materials within the endocrown design (p = 0.445).

When comparing restoration designs within each material, overlays demonstrated significantly higher fracture resistance than endocrowns for Crios (p = 0.004), whereas this difference was not statistically significant forTessera (p = 0.068).

The magnitude of observed differences was further evaluated using mean differences and corresponding 95% confidence intervals, providing an estimate of the practical relevance of the findings beyond statistical significance Table 2.

**Table 2:** Comparisons and summary statistics of fracture resistance (N) for different materials and designs

Material Design	Fracture resistance (N) (Mean ± SD)		*p*-value
	**Tessera**	**Crios**	
Endocrown	1114.88 ± 318.88	1354.20 ± 346.88	**0.445 ns**
Overlay	1712.33 ± 559.59	2384.60 ± 631.10	0.043*
*p*-value	**0.068 ns**	0.004*	

*ns* not significant

^*^Significant

**Fig. 5:**
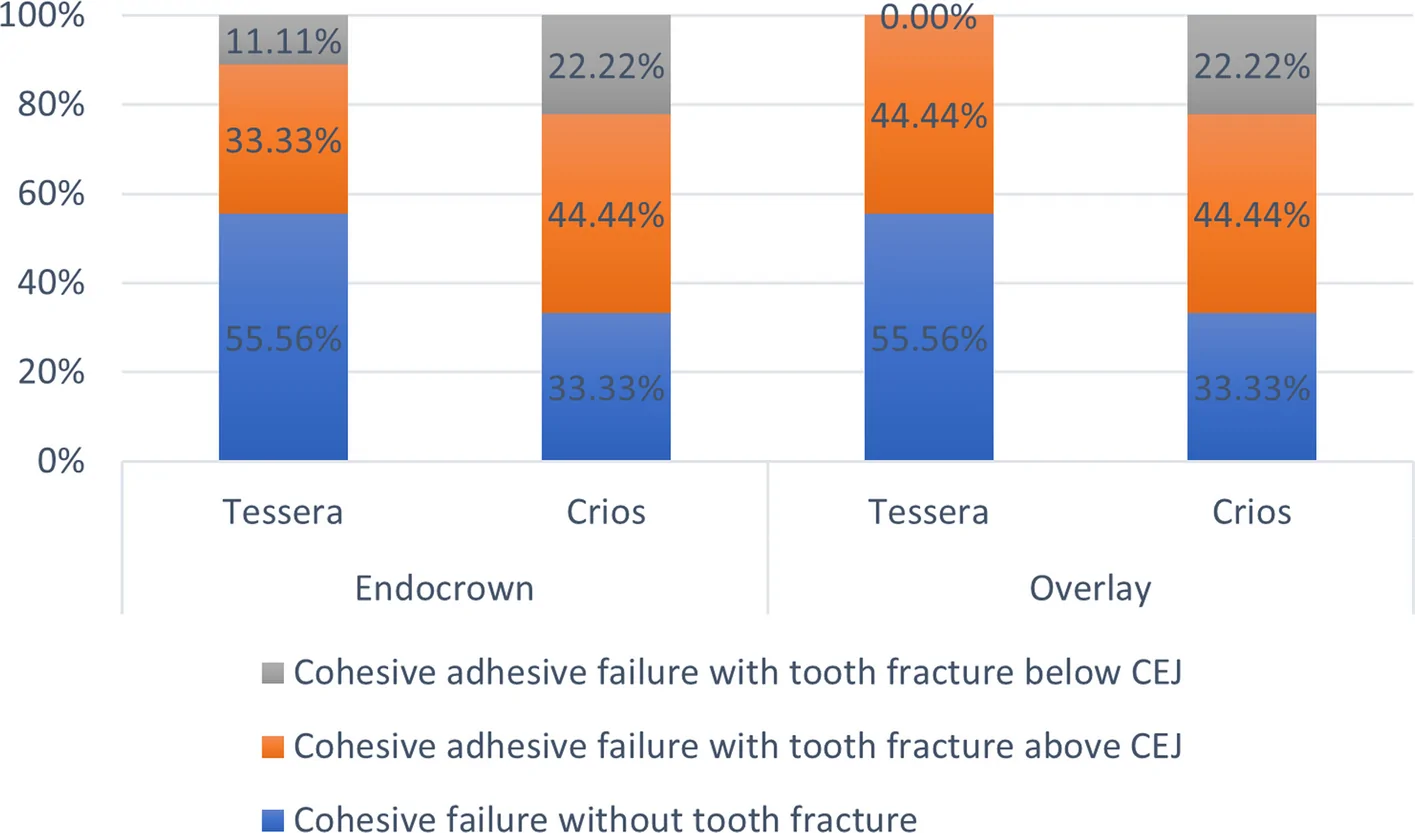
Stacked bar chart showing failure mode distribution

Failure modes revealed distinct patterns based on design and material. For endocrowns, cohesive failure without tooth fracture was most frequent in Tessera (55.56%) and Crios (33.33%), while cohesive adhesive failure with tooth fracture above the CEJ was the dominant mode for Crios (44.44%). In overlays, cohesive failure without tooth fracture remained the most common mode for Tessera (55.56%) and Crios (33.33%), with cohesive adhesive failure above the CEJ equally prevalent in both materials (44.44%). Failures below the CEJ were rare in Tessera and more frequent in Crios for both designs (Fig. 5).

## Discussion

The present in-vitro study evaluated the influence of restoration design (endocrown versus overlay) and CAD/CAM material (lithium disilicate versus indirect composite) on fracture resistance and failure modes of endodontically treated mandibular molars. The null hypothesis was partially rejected, as restoration design significantly affected fracture resistance, whereas material type showed a design-dependent influence.

### Effect of restoration design

Overlay restorations demonstrated significantly higher fracture resistance than endocrowns, irrespective of the CAD/CAM material used. This finding can be attributed to the biomechanical advantages of the overlay design, which more closely replicates the structural behavior of natural teeth by providing cuspal coverage and distributing occlusal forces over a broader surface area [21–24]. The presence of a fiber-reinforced composite core beneath the overlay further contributes to stress modulation by acting as a dentin analogue, allowing more uniform load transfer and reducing stress concentration at the adhesive interface and internal line angles [20, 25].

In contrast, endocrowns function as monoblock restorations anchored primarily within the pulp chamber. While this design is conservative and clinically successful in many scenarios, it may concentrate stresses within a single rigid structure, particularly under axial loading, which can predispose the restoration–tooth complex to earlier failure when compared with partial-coverage designs incorporating a compliant substructure [26, 27].

The superior performance of overlays observed in this study is consistent with several previous investigations. Zohdy et al. [28] reported higher fracture resistance values for ceramic overlays supported by resin cores compared with endocrowns, even when differences did not always reach statistical significance. Hezavehi et al. [29] similarly demonstrated that overlays exhibited higher fracture strength than endocrowns when fabricated from identical materials. These findings collectively support the concept that restoration design plays a decisive role in determining mechanical behavior, particularly in molars subjected to high occlusal loads.

Conversely, some studies have reported superior performance of endocrowns over overlays. For example, Veselinova et al. [30] and Amjadi et al. [31] found higher fracture resistance for endocrowns, particularly when restorations were fabricated from zirconia or when teeth were decoronated close to the cemento-enamel junction. The discrepancy between these findings and the present results can be explained by key methodological differences, including preparation geometry, remaining coronal tooth structure, restorative materials, and core design. In the present study, standardized MOD preparations with preserved cervical enamel were used, and overlays were fabricated on a flat fiber-reinforced composite base rather than filling the pulp chamber entirely. Such conservative preparation geometry may favor overlays by enhancing stress distribution and preserving tooth structure.

### Effect of restorative material

Regarding material effects, indirect composite (Crios) exhibited higher fracture resistance values than lithium disilicate (Tessera), reaching statistical significance in the overlay groups only. This behavior may be explained by the dentin-like elastic modulus of resin-matrix composites, which allows limited elastic deformation and enhanced energy absorption under functional loading [15, 17, 32].

The microstructure of resin-matrix composites promotes crack deflection and stress redistribution, which may delay catastrophic failure compared with brittle glass–ceramics [17, 32]. In contrast, lithium disilicate ceramics, despite their high flexural strength, remain susceptible to rapid crack propagation once critical flaws are activated [15].

The design-dependent influence of material observed in this study suggests that the mechanical advantages of resin-matrix composites are most pronounced when combined with overlay restorations, where stress distribution is more favorable. Similar findings were reported by Mertsöz et al. [33], who demonstrated superior fracture resistance for resin-matrix composite restorations compared with lithium disilicate endocrowns. Comparable trends have been reported in other in-vitro investigations evaluating partial-coverage restorations fabricated from resin-based materials [21, 23].

### Failure mode analysis

Failure mode analysis provides insight into the clinical implications of fracture behavior beyond load-to-failure values. In the present study, most failures were confined to the restoration or occurred above the cemento-enamel junction, which is generally considered a favorable outcome from a reparability standpoint [23, 27].

However, a higher incidence of sub-CEJ fractures was observed in the Crios groups compared with the Tessera groups. This finding suggests that while resin-matrix composites can absorb higher loads, their greater compliance may transmit stresses to the underlying tooth structure once critical loading thresholds are exceeded [20, 25]. Conversely, the higher stiffness of lithium disilicate ceramics may result in restoration-confined failures, effectively acting as a sacrificial layer that protects the remaining tooth structure [15, 32].

### Limitations

The limitations of this in-vitro study should be acknowledged. The use of extracted human teeth may not fully replicate the biological conditions present in vivo, such as periodontal ligament behavior, bone support, and neuromuscular feedback, which can influence stress distribution and failure patterns. Thermomechanical aging simulated early clinical service but does not represent long-term fatigue behavior [34, 35].

The present study did not include an intact or unrestored tooth control group, as the primary aim was to compare the mechanical performance of different restorative designs and materials for endodontically treated molars rather than to evaluate absolute fracture resistance relative to sound teeth. Consequently, the findings should be interpreted as comparative outcomes within the tested groups. Additionally, the study focused on mandibular molars with standardized MOD preparations and evaluated only two restorative designs and materials. Axial loading does not fully replicate complex occlusal dynamics. Therefore, further long-term clinical and laboratory investigations are required to validate these findings.

## Conclusion

Within the limitations of this in-vitro study, the following conclusions could be withdrawn:

1. Overlay restorations with a fiber-reinforced composite base exhibited significantly higher fracture resistance values compared to endocrowns in restoring endodontically treated molars.
2. Indirect composite (Crios) showed superior fracture resistance compared to lithium disilicate (Tessera) in overlay restorations, while no significant difference was observed between the materials in endocrown restorations.
3. The combination of overlay restorations with indirect composite material (Crios) yielded the highest fracture resistance among the tested groups.
4. Crios overlay restorations demonstrated fracture resistance values comparable to lithium disilicate overlays, suggesting their potential suitability for posterior load-bearing applications; however, differences in failure mode indicate that lithium disilicate restorations may exhibit more favorable failure characteristics.

## Data Availability

The datasets generated and/or analyzed during the current study are available from the corresponding author upon reasonable request.

## Abbreviations

ETT: Endodontically Treated Teeth
CAD/CAM: Computer-Aided Design/Computer-Aided Manufacturing
HSD: Honestly Significant Difference
CEJ: Cemento-Enamel Junction
MOD: Mesio-Occluso-Distal
